# Pathway coessentiality mapping reveals complex II is required for de novo purine biosynthesis in acute myeloid leukaemia

**DOI:** 10.1038/s42255-025-01410-x

**Published:** 2025-12-05

**Authors:** Amy E. Stewart, Derek K. Zachman, Pol Castellano-Escuder, Lois M. Kelly, Ben Zolyomi, Michael D. I. Aiduk, Christopher D. Delaney, Ian C. Lock, Claudie Bosc, John Bradley, Shane T. Killarney, J. Darren Stuart, Paul A. Grimsrud, Olga R. Ilkayeva, Christopher B. Newgard, Navdeep S. Chandel, Alexandre Puissant, Kris C. Wood, Matthew D. Hirschey

**Affiliations:** 1 Department of Pharmacology & Cancer Biology, Duke University School of Medicine, Durham, NC USA; 2 Duke Molecular Physiology Institute and Sarah W. Stedman Nutrition and Metabolism Center, Duke University School of Medicine, Durham, NC USA; 3 Department of Pediatrics, Division of Hematology-Oncology, Duke University, Durham, NC USA; 4 INSERM U1342, IRSL, Saint-Louis Hospital, Paris Cité University, Paris, France; 5 Departments of Medicine and Robert H. Lurie Cancer Center, Northwestern University Feinberg School of Medicine, Chicago, IL USA; 6 Department of Biostatistics & Bioinformatics, Duke University School of Medicine, Durham, NC USA; 7 Department of Medicine, Division of Endocrinology, Metabolism, and Nutrition, Department of Medicine, Duke University, Durham, NC USA; 8 Cardiovascular and Metabolic Disorders Program, Duke-National University of Singapore (NUS) Medical School, Singapore, Singapore

**Keywords:** Acute myeloid leukaemia, Cancer metabolism, Metabolism, Metabolomics

## Abstract

Understanding how cellular pathways interact is crucial for treating complex diseases like cancer. Individual gene–gene interaction studies have provided valuable insights, but may miss pathways working together. Here we develop a multi-gene approach to pathway mapping which reveals that acute myeloid leukaemia (AML) depends on an unexpected link between complex II and purine metabolism. Through stable-isotope metabolomic tracing, we show that complex II directly supports de novo purine biosynthesis and that exogenous purines rescue AML cells from complex II inhibition. The mechanism involves a metabolic circuit where glutamine provides nitrogen to build the purine ring, producing glutamate that complex II metabolizes to sustain purine synthesis. This connection translates into a metabolic vulnerability whereby increasing intracellular glutamate levels suppresses purine production and sensitizes AML cells to complex II inhibition. In a syngeneic AML mouse model, targeting complex II leads to rapid disease regression and extends survival. In individuals with AML, higher complex II gene expression correlates with resistance to BCL-2 inhibition and worse survival. These findings establish complex II as a central regulator of de novo purine biosynthesis and a promising therapeutic target in AML.

## Abstract (web summary)

A machine-learning-based computational approach to probe pathway coessentiality reveals that complex II of the electron transport chain regulates de novo purine synthesis, and can be targeted to treat acute myeloid leukaemia.

## Main

Cells orchestrate their functions through connected networks of interacting genes. Coessentiality mapping reveals these networks by identifying genes that share similar patterns of essentiality across conditions^1–3^. While this approach has successfully uncovered important gene pairs^3,4^, scaling to genome-wide analysis for human genomic research presents nearly 200 million possible interactions. In contrast, mapping connections between gene pathways may provide an opportunity to organize coessentiality data into higher levels to identify associations less apparent with single gene–gene queries. Interrogating pathway-level interactions may be of particular interest in metabolism research, as a growing number of studies demonstrate that metabolic pathways connect to and regulate each other in unexpected ways. Specifically, mitochondrial electron transport chain (ETC) complexes have been shown to have critical roles in cancer by supporting amino acid^5–7^, nucleotide^8–10^ and epigenetic^11,12^ processes. Here, we developed a pathway coessentiality mapping tool to determine pathway–pathway interactions across the genome, focusing particularly on ETC components. This approach revealed an unexpected discovery: complex II (succinate dehydrogenase, SDH) directly regulates purine synthesis in acute myeloid leukaemia (AML), driving disease progression through this previously unknown metabolic link.

## Complex II has unique pathway associations

**Fig. 1:**
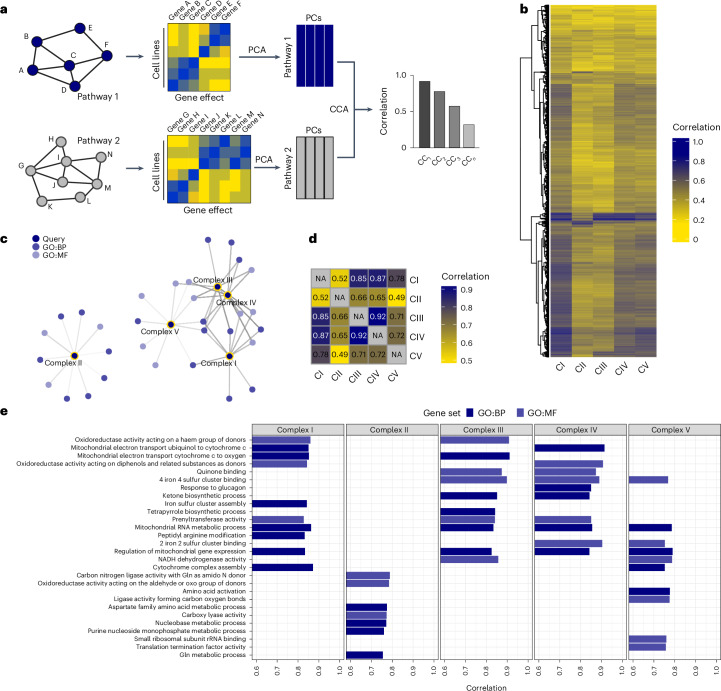
Pathway coessentiality mapping reveals a unique association between complex II and nucleotides. **a**, Development of pathway mapping model. Gene sets representing ~15,000 genes were used to select essentiality data across all cancer cell lines in the Cancer Dependency Map. For each gene set (depicted as ‘pathway’), the first four PCs were extracted from the gene dependency matrix and CCA was used to determine correlations between pathways. CC here refers to canonical correlation. **b**, Heat map of the canonical correlations between each ETC complex and 2,827 pathway gene sets. *k*-nearest-neighbours hierarchical clustering was used to group pathways by their coessentiality patterns (side). The colour of each cell represents the relative correlation strength for each pathway–complex comparison. **c**, Network diagram showing the connectedness of ETC complexes with GO pathways, with each ETC complex or pathway represented as a node and each connection as an edge. Notably, a larger sub-network includes complexes I, III, IV and V and related pathways, whereas a smaller sub-network includes only complex II and its unique pathways. **d**, The association between ETC complexes (consisting of both core and accessory subunits; Extended Data Fig. 1). The colour of each cell and the associated correlation score depict the strength of the complex–complex association. NA, not applicable. **e**, Top pathway associations for each ETC complex, with multiple shared pathways shown for complexes I, III, IV and V and distinct pathways for complex II. The correlation strength between each pathway and complex is shown at the bottom. rRNA, ribosomal RNA. For **c** and **e**, the colour of nodes or bars represents a GO pathway type as indicated. Panel **a** created with BioRender.com. Source Data

**Extended Data Fig. 1:**
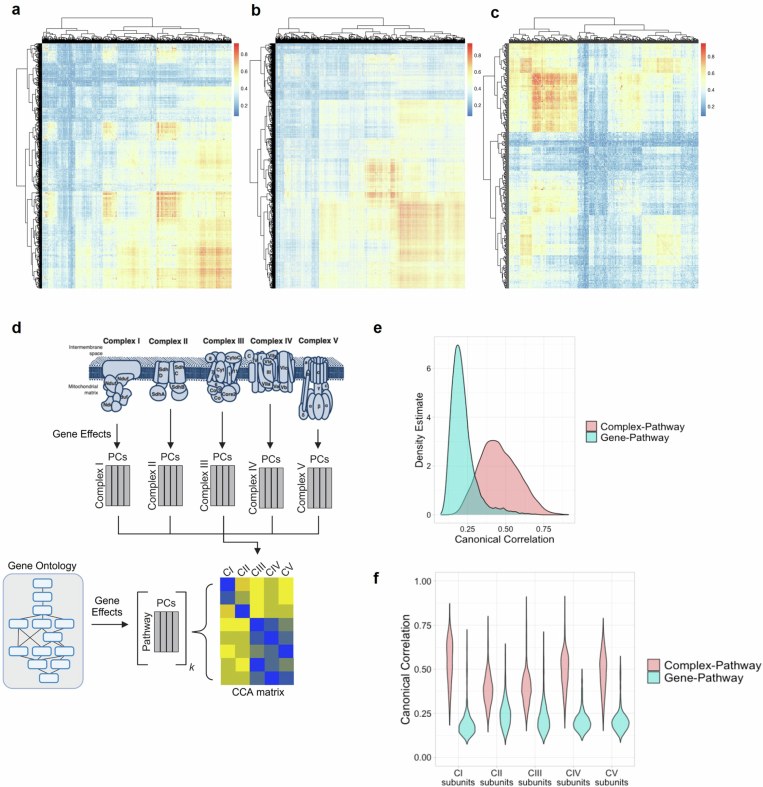
Pathway coessentiality mapping reveals new pathway interactions and strengthens existing associations. **a-c**) Heatmaps depicting pathway-pathway correlation matrices (with canonical correlations ranging 0-1) for the Gene Ontology categories of (**a**) Molecular Function, (**b**) Biologic Process, and (**c**) Cellular Component. **d**) Overview of the CCA pipeline to determine canonical correlations between ETC complexes and GO pathways. Genes comprising ETC complexes or GO pathways were first represented by PCs, and then the CCA was calculated for each complex-pathway pair. The term Gene Effects designates the use of Dependency Scores from DepMap. Created with BioRender.com. **e**) Overall distribution comparing ETC complex-pathway and ETC gene-pathway values for all ETC-pathway correlations. Pink plots show correlation values when comparing pathways to ETC complexes using PC extraction of ETC genes. Teal plots show the correlations for individual ETC genes with pathways. **f**) Violin plot showing the effect of ETC PC embeddings on the correlation strengths between each ETC complex and all pathways. Correlation strength was consistently stronger when PCs were first extracted to represent ETC genes as complexes. Source Data

To map pathway-level connections, we analysed nearly 3,000 gene sets from multiple annotation systems: canonical pathways, Gene Ontology (GO) pathways, transcription factor targets and cell-type signatures. These sets encompassed approximately 15,000 expressed genes^13^. To evaluate pathway-level connections, we developed a systematic approach using CRISPR-based dependency scores from the Cancer Dependency Map^14^. First, we extracted the top principal components (PCs) for each pathway. Then, we used canonical correlation analysis (CCA)^15^ to find maximum correlations between pathway PCs (Fig. 1a). This revealed distinct patterns of pathway coessentiality across major biological domains—biological process (BP), molecular function (MF) and cellular components (CC) (Extended Data Fig. 1a–c).

The ETC served as an ideal test case for this approach. Its protein complexes coordinate electron transport and hydrogen pumping with oxidative phosphorylation (OxPhos) to support various biosynthetic functions^5,6^. We analysed how each of the complexes I–V connected to cellular pathways by examining their subunits collectively (Extended Data Fig. 1d). Importantly, embedding ETC genes as complexes yielded overall stronger coessentiality scores than when considering ETC genes individually (Extended Data Fig. 1e,f and Supplementary Table 1). Comparing the coessentiality patterns of the ETC complexes with cellular pathways, hierarchical clustering revealed complex II stood apart from the other ETC components (Fig. 1b). Whereas complexes III and IV showed strong similarity in their pathway connections, as did complexes I, III, IV and V—matching their known ability to form supercomplexes^16^—complex II showed a unique pattern. Network visualization reinforced this finding, showing that complexes I, III, IV and V formed a tight cluster through shared coessential pathways, while complex II and its associated pathways formed a separate, distinct network (Fig. 1c). This distinction appeared both in pathway associations and in direct ETC gene coessentiality analysis, where complex II showed weak associations with other ETC complexes (Fig. 1d).

Complex II oxidizes succinate while reducing ubiquinone^17^. Our computational analysis revealed 31 cellular pathways with unique complex II connectivity (Supplementary Table 2). The strongest connections emerged with nucleotide and amino acid metabolism—specifically the ‘nucleobase metabolic process’, ‘purine nucleobase monophosphate metabolic process’ and ‘glutamine (Gln) metabolic process’. No other ETC complex shared these metabolic connections (Fig. 1e). This distinct pathway signature pointed to a previously unrecognized role for complex II in cancer cell metabolism via the potential regulation of nucleotide biosynthesis.

## Complex II is essential for haematological malignancies including AML

**Fig. 2:**
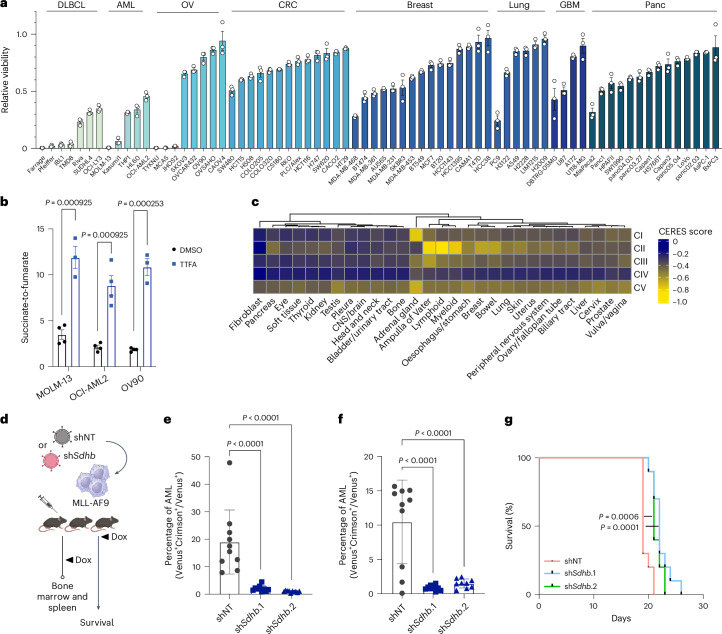
Complex II is essential for AML. **a**, Relative viability for 72 cancer cell lines after 3 days of complex II inhibition with TTFA. Cell lines are grouped by cancer cell lineage according to Cancer Cell Line Encyclopedia annotations. OV, ovarian; CRC, colorectal carcinoma; GBM, glioblastoma multiforme; Panc, pancreatic. Data shown are the mean ± s.e.m. for *n* = 3 replicates. **b**, Metabolite ratio of succinate to fumarate after 24 h in control conditions (*n* = 4 replicate experiments) or with complex II inhibition (*n* = 3–4 replicate experiments) in two AML (MOLM-13, OCI-AML2) cell lines sensitive to TTFA and an ovarian cancer cell line (OV90) insensitive to TTFA. **c**, Heat map of dependency (CERES) scores for each ETC complex across cancer lineages as represented in the Cancer Dependency Map. Dependency scores are expressed as the mean score for ETC genes in all cells within a given lineage, with a more negative number indicating a stronger dependency. CNS, central nervous system. **d**, Experimental model for *Sdhb* knockdown in the MLL-AF9-driven AML mouse model. Cells were infected with retroviral vectors expressing doxycycline-inducible non-targeting (shNT) or one of two *Sdhb*-targeting (sh*Sdhb*.1 or sh*Sdhb*.2) shRNA and transplanted into recipient mice for bone marrow, spleen or survival analysis. **e**, Bone marrow AML burden after doxycycline treatment in the MLL-AF9 model. AML actively expressing MLL-AF9 and sh*Sdhb* or shNT (Venus^+^Crimson^+^) were compared to all cells expressing MLL-AF9 (Venus^+^). **f**, Spleen AML burden after doxycycline treatment. Spleen AML content is expressed as in **e**. **g**, Survival of MLL-AF9 leukaemic mice after shNT or sh*Sdhb* induction with doxycycline (*n* = 10 mice per treatment group for each experiment in **e**–**g**). All data are presented as the mean ± s.e.m. unless otherwise indicated. *P* values were determined by one-way analysis of variance (ANOVA; **b**,**e**,**f**) or by a log-rank test (**g**). Panel **d** created with BioRender.com. Source Data

**Extended Data Fig. 2:**
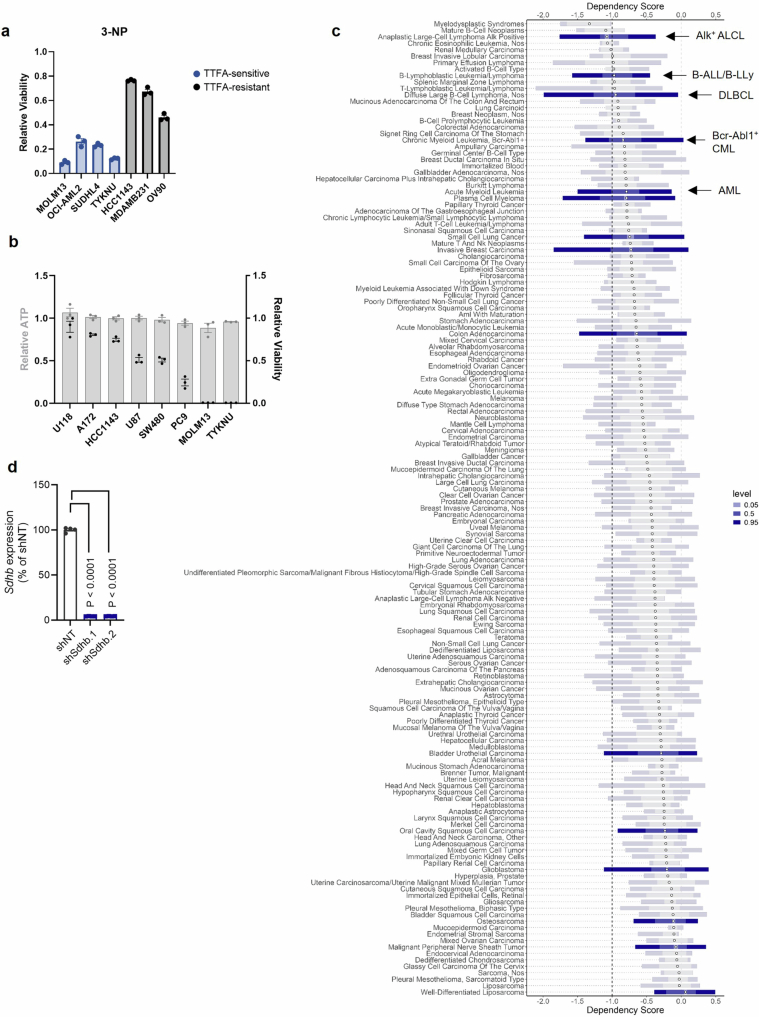
Sensitivity of leukaemia and lymphoma cancer subtypes to complex II disruption. **a**) Effect of Complex II inhibitor 3NP (2 mM) on viability of select cell lines that are either sensitive or resistant to TTFA. **b**) Differential effect of Complex II inhibition by TTFA on ATP levels (grey bars) assayed after 30 minutes compared to viability (black squares) recorded after 3 days in culture with TTFA (viability values used from experiments in Fig. 2a). Data in (**a-b**) show mean ± s.e.m. from n = 3 independent experiments. **c**) Complex II sub-lineage dependencies in cancer. Horizontal bars represent ranges of dependency scores for cancer sub-lineages, with differential Complex II dependency indicated by solid blue versus transparent gray bars. Shades of blue or gray within each bar represent the proportion of cells with a given dependency score interval, annotating 5%, 50% and 95% of cells within each sub-lineage (indicated in the legend as ‘level’). Leukemia and lymphoma subtypes with dependency scores that are significantly different from the aggregate of other sub-lineages are shown with arrows (Alk^+^ ALCL, Alk^+^ anaplastic large cell lymphoma; B-ALL/B-LLy, B-cell acute lymphoblastic leukemia/B-cell lymphoblastic lymphoma; DLBCL, diffuse large B-cell lymphoma; Bcr-Abl1^+^ CML, Bcr-Abl1^+^ chronic myeloid leukemia; AML, acute myeloid leukemia). **d**) *Sdhb* gene expression in the MLL-AF9 AML murine model after doxycycline induction of non-targeting (shNT) or *Sdhb*-targeting (*Sdhb*.1, *Sdhb*.2) small hairpin RNAs. Data show mean ± s.e.m. from n = 4 independent experiments. P values were calculated using a one-way ANOVA with a Tukey’s post-hoc test. Source Data

To determine which cancers rely on complex II activity, we first screened a panel of 72 diverse cancer cell lines for sensitivity to complex II inhibition. We used 2-thenoyltrifluoroacetone (TTFA), a potent complex II inhibitor, at a dose (200 μM) that effectively blocks complex II activity^18^. Cancer cell lines derived from the haematological malignancies, including AML and diffuse large B cell lymphoma (DLBCL), stood out as most sensitive to complex II inhibition (Fig. 2a). We confirmed the sensitivity profile of a subset of cancer cell lines with an additional inhibitor, 3-nitroproprionic acid (3-NP), to ensure that results were representative of on-target complex II inhibition (Extended Data Fig. 2a). The requirement for complex II activity was not driven by differences in target inhibition, as measured by changes to the succinate-to-fumarate ratio or by adenosine triphosphate (ATP) loss (Fig. 2b and Extended Data Fig. 2b).

Next, we analysed data from DepMap to more broadly assess ETC complex dependency across cancers. Consistent with our chemical screen, this revealed that blood cancers have greater sensitivity to complex II genetic disruption (Fig. 2c). Specifically, both myeloid and lymphoid cancers depend strongly on complex II genes for survival. Further examination of specific cancer subtypes highlighted complex II’s critical role in multiple types of leukaemias and lymphomas, including AML and related diseases such as myelodyplastic syndromes (Extended Data Fig. 2c). These results corroborated our chemical screen showing that blood cancers, including AML, have a specific vulnerability to complex II inhibition that distinguishes them from other cancer types.

Complex II function requires its catalytic subunit *SDHB*, which handles Fe-S-mediated electron transfer to ubiquinone. To test whether this requirement is observed in physiological mouse models, we examined complex II loss in a syngeneic MLL-AF9-driven mouse model of AML (Fig. 2d). This model replicates human AML driven by the t(9;11)(p22;q23) translocation in blood stem cells^19^. We depleted *Sdhb* through doxycycline-induced shRNA, which potently reduced *Sdhb* gene expression (Extended Data Fig. 2d). Remarkably, loss of *Sdhb* expression reduced AML burden in mouse bone marrow and spleen and significantly extended survival (Fig. 2e–g). These data demonstrate complex II is essential for AML in vitro and in vivo.

## Complex II regulates purines to maintain AML survival

**Fig. 3:**
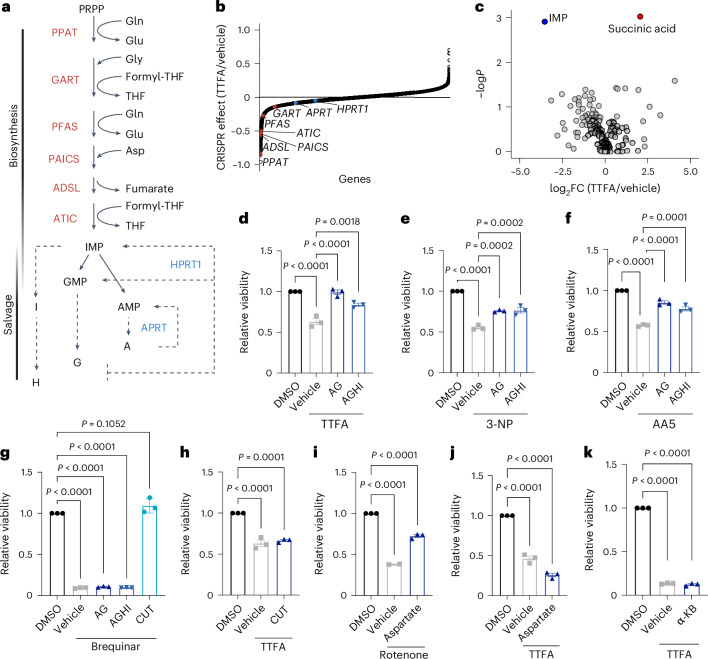
Complex II regulates purine levels to maintain AML survival. **a**, Schematic of the de novo and salvage purine synthesis pathways beginning with PRPP and ending with IMP, adenosine monophosphate (AMP) and guanosine monophosphate (GMP). Enzymes of the de novo biosynthesis pathway are shown in red, and enzymes mediating purine salvage are shown in blue. **b**, Waterfall plot showing the effect of CRISPR loss-of-function mutations on the sensitivity of OCI-AML2 cells to complex II inhibition. Purine de novo biosynthesis genes are shown in red, and purine salvage genes are shown in blue. The effect of common essential cancer genes was removed. **c**, Volcano plot showing the effect of complex II inhibition on steady-state metabolite levels in MOLM-13 cells. Two-tailed *t*-tests were used to compare metabolites and raw *P* values are shown. **d**–**f**, Effect of exogenous purine add-back of A, G, H or I (each 30 μM) on AML viability following complex II inhibition with TTFA (**d**), 3-NP (**e**) or AA5 (**f**) in OCI-AML2 cells. **g**, Effect of purine or pyrimidine add-back on OCI-AML2 cell viability following pyrimidine synthesis inhibition by brequinar (2 μM). Pyrimidines are cytidine (C), uridine (U) and thymidine (T) (each 30 μM). **h**, Effect of pyrimidine add-back on viability following complex II inhibition by TTFA in OCI-AML2 cells. **i**, Effect of aspartate add-back (20 mM) on the viability of MOLM-13 cells when cultured in control (DMSO group) conditions or with the complex I inhibitor rotenone. **j**,**k**, Effect of either aspartate (**j**) or α-KB (1 mM; **k**) add-back on the viability of MOLM-13 cells cultured in DMSO or TTFA. For all plots, data are shown as the mean ± s.e.m. of *n* = 3 independent experiments unless otherwise noted. *P* values were determined by one-way ANOVA with Tukey’s post hoc test (**d**–**k**). Panel **a** created with BioRender.com. Source Data

Our pathway coessentiality analysis identified specific metabolic pathways linked to complex II, including purine nucleotides (Fig. 1e and Supplementary Table 2). Purines are synthesized de novo by the purinosome, a six-enzyme metabolon that localizes to the periphery of mitochondria during purine starvation^20^. Alternatively, purines can be regenerated via the salvage pathway (Fig. 3a). To provide an orthogonal validation for the link between complex II and nucleotide metabolism, we performed loss-of-function CRISPR screening in OCI-AML2 cells undergoing complex II inhibition by TTFA. This unbiased approach confirmed that among ~16,000 genes, loss of nucleotide biosynthesis enzymes strongly sensitized AML cells to complex II inhibition (Fig. 3b and Supplementary Table 3).

**Extended Data Fig. 3:**
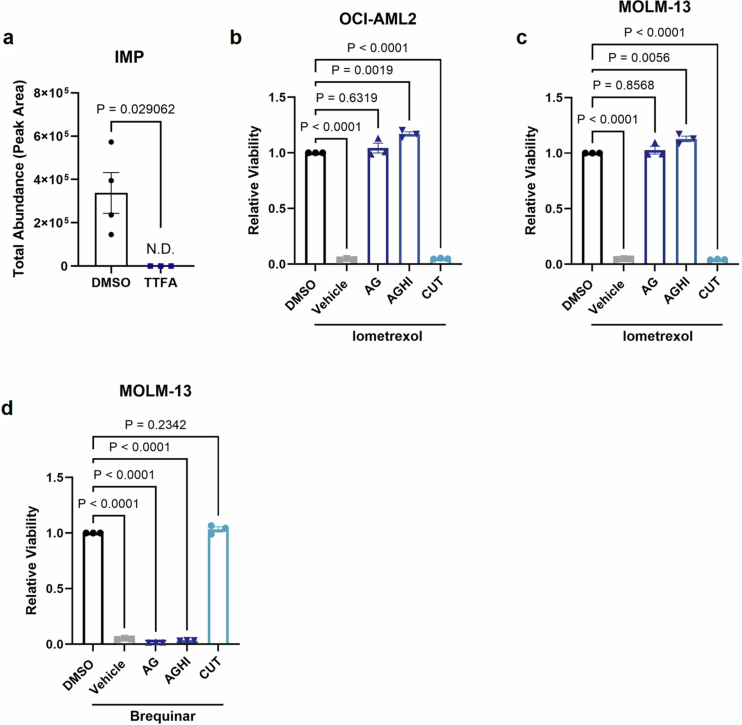
Nucleotide salvage is active in AML cells. **a**) Effect of Complex II inhibition by TTFA on the steady state level of *de novo* purine biosynthesis product IMP. **b-c**) Effect of exogenous purines (adenine (**A**), guanine (**G**), hypoxanthine (**H**), inosine (**I**)) or pyrimidines (cytidine (**C**), uridine (**U**), thymidine (**T**)) on viability in (**b**) OCI-AML2 cells or (**c**) MOLM-13 cells treated with the targeted purine synthesis (GART) inhibitor, lometrexol. Each nucleotide species was added back at a concentration of 30 μM. **d**) Effect of exogenous purines or pyrimidines on viability of MOLM-13 cells treated with the pyrimidine synthesis inhibitor, brequinar (2 μM). All data in show mean ± s.e.m. from n = 3 independent experiments. P values were calculated using unpaired, two-tailed Student’s t-test (**a**) or a one-way ANOVA with a Tukey’s post-hoc test (**b-d**). Source Data

To explore the metabolic pathways that emerged as uniquely coessential with complex II, we performed liquid chromatography–mass spectrometry (LC–MS) metabolic profiling. Complex II inhibition increased succinate levels, the succinate-to-fumarate ratio (Figs. 2b and 3c) and reduced de novo purines including inosine monophosphate (IMP; Fig. 3c, Extended Data Fig. 3a and Supplementary Table 4). This suggested that AML’s sensitivity to complex II inhibition stems from purine depletion. The purine salvage pathway can compensate for reduced purine synthesis by converting purine bases—adenine (A), guanine (G), hypoxanthine (H) and inosine (I)—into their respective nucleotides (Fig. 3a). Previous studies showed that purine salvage can rescue cells from purine synthesis inhibitors^21,22^. Further, complex I inhibition activates the purine salvage pathway through *HPRT1* (ref. ^8^). Thus, we tested if supplementation with purines (AG or AGHI) rescued cells from complex II inhibition by TTFA. First, we established that purines rescued cell growth from treatment with the purine synthesis inhibitor lometrexol (LTX; Extended Data Fig. 3b,c). Remarkably, external purines also rescued AML cells from complex II inhibition by TTFA (Fig. 3d). This rescue extended to two structurally distinct complex II inhibitors, 3-NP and atpenin A5 (AA5; Fig. 3e,f). However, pyrimidines failed to rescue cells from TTFA treatment despite their effectiveness against pyrimidine synthesis inhibition by brequinar (Fig. 3g,h and Extended Data Fig. 3d). These results establish that complex II maintains AML survival specifically through purine regulation.

Previous work showed that in osteosarcoma cells, aspartate or the electron acceptor α-ketobutyrate (α-KB) can rescue complex II inhibition by AA5 (ref. ^23^). Unlike complex I inhibition using rotenone, which aspartate can rescue (Fig. 3i), neither aspartate nor α-KB rescued AML cells from complex II inhibition (Fig. 3j,k). This ruled out aspartate deficiency as the cause of purine suppression in AML.

## Complex II supports de novo purine synthesis

**Fig. 4:**
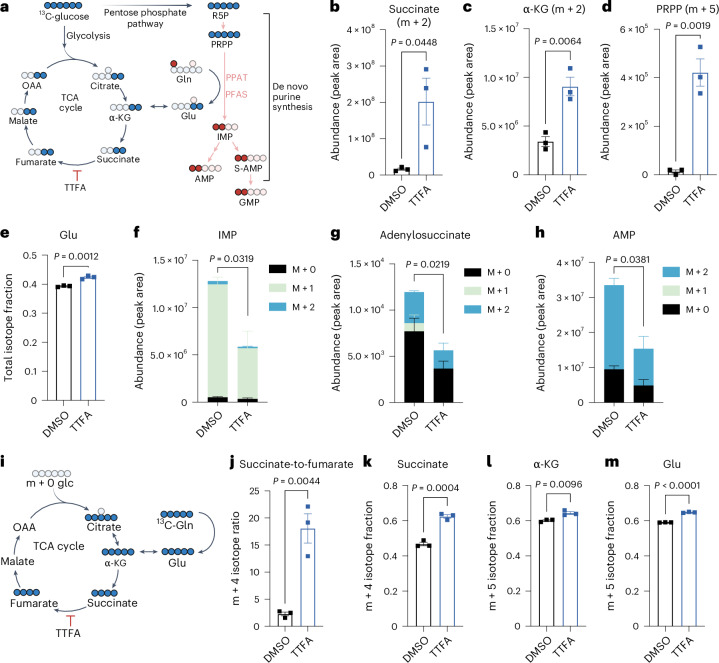
Complex II supports de novo purine biosynthesis. **a**, Overview of metabolic pathways interrogated with stable-isotope tracing studies using ^13^C_6_-glucose or ^15^N_1_-Gln. Relevant metabolites for ^13^C_6_-glucose tracing are shown in blue, and the relevant metabolites for ^15^N_1_-Gln tracing are shown in red. S-AMP is also known as adenylosuccinate. **b**,**c**, Effect of complex II inhibition on ^13^C_6_-glucose-derived carbon in m + 2 succinate (**b**) and m + 2 α-KG (**c**). **d**, Effect of complex II inhibition on ^13^C_6_-glucose-derived m + 5 PRPP. **e**, Effect of complex II inhibition on the ^13^C_6_-glucose-derived isotope fraction of Glu. **f**–**h**, Effect of complex II inhibition on the abundance of ^15^N_1_-Gln nitrogen incorporation into de novo purines including IMP (**f**), adenylosuccinate (**g**) and AMP (**h**). **i**, Overview of possible routes of carbon distribution in oxidative or reductive TCA cycle reactions following anaplerosis of ^13^C_5_-Gln. **j**–**m**, Effect of complex II inhibition on ^13^C_5_-Gln carbon for the following: m + 4 succinate-to-m + 4 fumarate ratio (**j**), m + 4 succinate isotope fraction (**k**), m + 5 α-KG isotope fraction (**l**) and m + 5 Glu isotope fraction (**m**). Results show the mean ± s.e.m. for *n* = 3 independent experiments. *P* values are shown for unpaired, two-tailed Student’s *t*-tests. In **f**–**h**, the *P* values reported are for the sum of all isotopes. OAA, oxaloacetate. Panels **a** and **i** created with BioRender.com. Source Data

**Extended Data Fig. 4:**
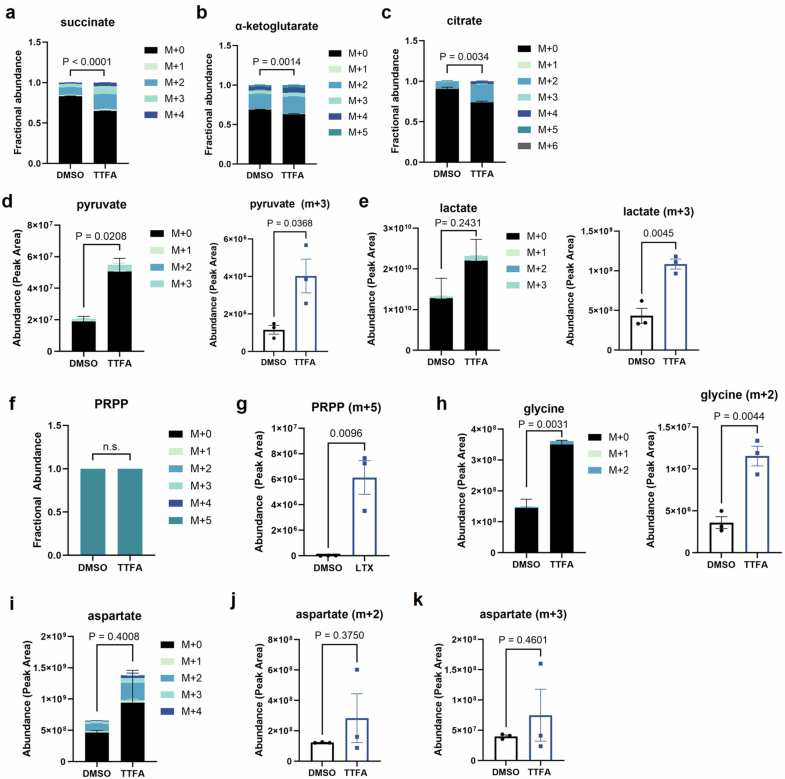
Complex II inhibition disrupts central carbon metabolism. **a-c**) Effect of Complex II inhibition on the fractional composition of (**a**) succinate, (**b**) α-ketoglutarate, and (**c**) citrate from ^13^C_6_-glucose tracing studies. P values are comparing the total isotope fraction between DMSO and TTFA conditions. **d-e**) Effect of Complex II inhibition on ^13^C_6_-glucose-derived isotope content of glycolytic metabolites (**d**) pyruvate and (**e**) lactate. For each, the left plot shows abundance for all isotopes, and the right plot shows the m + 3 isotope. **f**) Effect of Complex II inhibition on the fractional abundance of ^13^C_6_-glucose-derived PRPP. **g**) Quantification of ^13^C_6_-glucose-derived m + 5 PRPP after culture in the presence or absence of the purine synthesis inhibitor, lometrexol (LTX). **h**) Effect of Complex II inhibition on the ^13^C_6_-glucose-derived isotope content in glycine. The left plot shows the abundance of all isotopes, and the right plot shows abundance for the m + 2 isotope. **i-k**) Effect of Complex II inhibition on ^13^C_6_-glucose derived aspartate isotopes, including (**i**) total peak area for aspartate, (**j**) peak area for m + 2 aspartate and (**k**) m + 3 aspartate, which are derived from oxidative and reductive pyruvate metabolism, respectively. For all panels, data shown are mean ± s.e.m. for n = 3 replicate experiments. P values were calculated using unpaired, two-tailed Student’s t-tests. For figure panels showing the peak area of multiple isotopes, the P values are reported for the total abundance. Source Data

Next, we used stable-isotope tracing with labelled glucose or Gln to uncover how complex II influences purine metabolism (Fig. 4a). Complex II inhibition caused striking metabolic changes. First, ^13^C_6_-glucose-derived carbon accumulated in succinate and upstream tricarboxylic acid (TCA) cycle metabolites (α-ketoglutarate (α-KG) and citrate), indicating blocked oxidative glucose metabolism (Fig. 4b,c and Extended Data Fig. 4a–c). This blockade forced glucose-derived carbon into alternative pathways, increasing glycolytic metabolites pyruvate and lactate (Extended Data Fig. 4d,e).

Glucose enters purine synthesis through the pentose phosphate pathway. This pathway produces phosphoribosyl pyrophosphate (PRPP), which forms the sugar backbone for all nucleotides. Given our observation that TTFA treatment reduces purine levels (Fig. 3c), we examined PRPP metabolism. Both complex II inhibition (TTFA) and direct purine synthesis inhibition (LTX) caused glucose-derived PRPP accumulation (Fig. 4d and Extended Data Fig. 4f,g). Complex II inhibition also increased glucose-derived carbon in other purine building blocks such as glycine (Extended Data Fig. 4h). Notably, both oxidatively derived aspartate (m + 2) and pyruvate carboxylase-derived aspartate (m + 3) levels stayed constant with TTFA treatment (Extended Data Fig. 4i–k). These results demonstrate that blocking complex II causes purine precursors to accumulate while final purine products decline.

**Extended Data Fig. 5:**
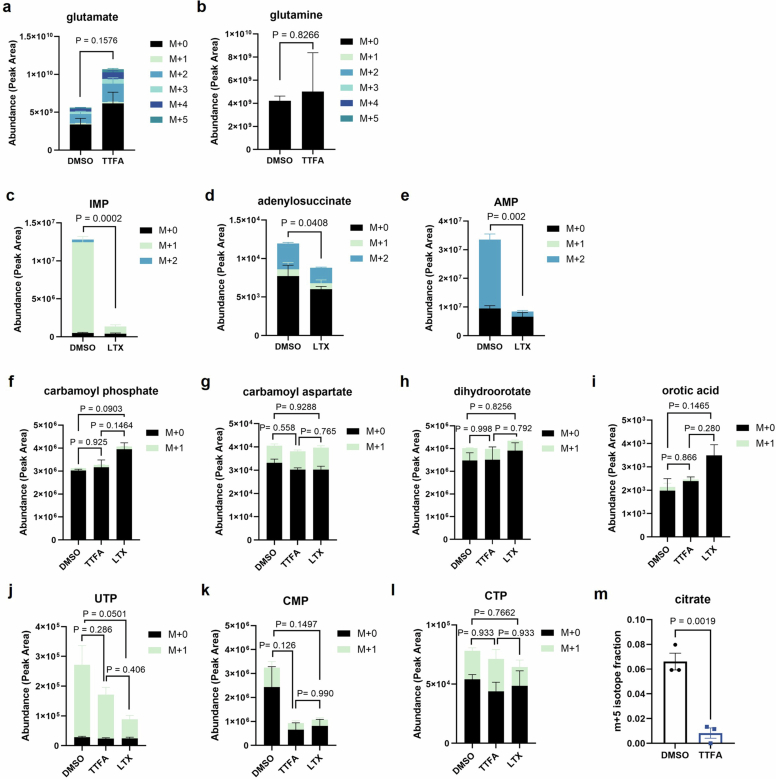
Impact of complex II or direct purine synthesis inhibition on metabolite isotope tracing from ^13^C_6_-glucose or ^15^N_1_-glutamine. **a**) Effect of Complex II inhibition on ^13^C_6_-glucose-derived glutamate isotope abundance. **b**) Effect of Complex II inhibition on ^13^C_6_-glucose-derived glutamine isotope abundance. **c-e**) Effect of LTX on the incorporation of ^15^N_1_-glutamine into *de novo* purines (**c**) IMP, (**d**) adenylosuccinate and (**e**) AMP. **f-l**) Effect of either Complex II inhibition (TTFA) or LTX on ^15^N_1_-glutamine derived nitrogen into pyrimidines. Abbreviations: UTP, uridine triphosphate; CMP, cytidine monophosphate; CTP, cytidine triphosphate. **m**) Effect of Complex II inhibition on the fractional content of m + 5 citrate derived from ^13^C_5_-glutamine. All data are shown as mean ± s.e.m. of n = 3 independent experiments. P values were calculated using unpaired, two-tailed Student’s t-tests (**a-e, m**) or a one-way ANOVA with Tukey’s post-hoc test (**f-l**). For metabolites with multiple isotopes, the P values are reported for the total abundance. Source Data

Gln emerged as a key player in this metabolic circuit. Our ^13^C_6_-glucose tracing revealed complex II inhibition caused glutamate (Glu) accumulation while Gln levels remained stable (Fig. 4e and Extended Data Fig. 5a,b). This aligned with our earlier finding of a computational link between complex II and Gln metabolism (Fig. 1c–e). Using ^15^N_1_-Gln-amide tracing, we confirmed that complex II inhibition specifically impaired Gln nitrogen donation to purines, shown by decreased m + 1 and m + 2 isotopes of IMP, AMP and adenylosuccinate (Fig. 4f–h). This matched the effect of direct purine synthesis inhibition with LTX (Extended Data Fig. 5c–e). Importantly, pyrimidine synthesis remained largely unaffected (Extended Data Fig. 5f–l), consistent with the inability of exogenous pyrimidines to rescue complex II inhibition in AML (Fig. 3h).

While some OxPhos mutations increase Gln anaplerosis into the TCA cycle^24^, complex II inhibition had the opposite effect. Using ^13^C_5_-Gln tracing (Fig. 4i), we found that TTFA treatment blocked Gln carbon metabolism by the TCA cycle. This caused accumulation of m + 4 succinate, m + 5 α-KG and m + 5 Glu, as well as an increased m + 4 succinate-to-m + 4 fumarate ratio (Fig. 4j–m). Complex II inhibition also reduced Gln’s reductive carboxylation, shown by decreased m + 5 citrate (Extended Data Fig. 5m). In sum, these data demonstrate that complex II inhibition creates a metabolic bottleneck by blocking carbon flow through the TCA cycle while simultaneously disrupting Gln’s critical nitrogen donation to purine synthesis.

## Glu accumulation upstream of complex II suppresses purines and is toxic to AML

**Fig. 5:**
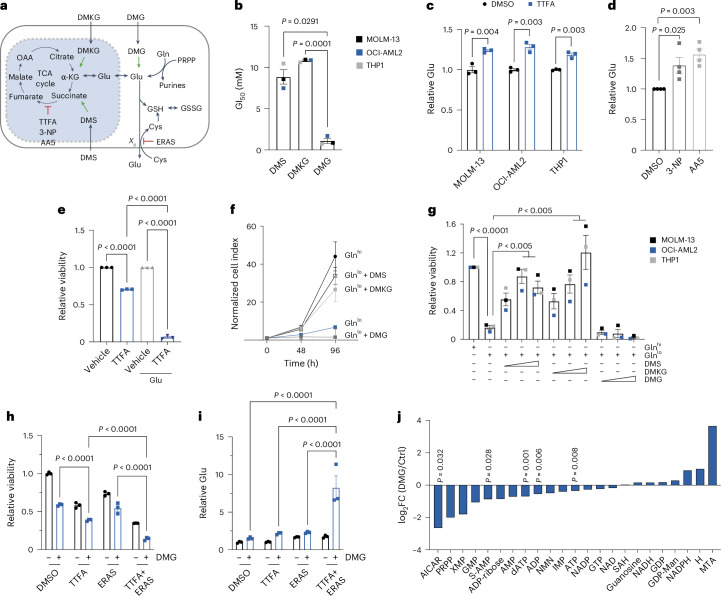
Glu accumulation upstream of complex II inhibits AML and reduces purine levels. **a**, Diagram showing metabolic pathways upstream of complex II, highlighting that Glu produced from de novo purine biosynthesis can be used for TCA cycle anaplerosis. Alternatively, Glu can be exported through system *X*_*c*_ (which is targeted by ERAS) or used for glutathione biosynthesis that is coupled to cystine (Cys) import. GSH, reduced glutathione; GSSG, glutathione disulfide. **b**, Effects of DMS, DMKG or DMG on growth of AML cell lines. GI_50_ values for each cell line were calculated from *n* = 3 replicate experiments. *P* values were calculated using one-way ANOVA with Tukey’s post hoc test. **c**, Relative intracellular Glu content in AML cells treated with TTFA (200 μM) for 24 h (*n* = 3 replicates). *P* values were calculated using two-way ANOVA with Tukey’s post hoc test. **d**, Relative intracellular Glu content in OCI-AML2 cells treated with 3-NP (1 mM) or AA5 (500 nM) for 48 h (*n* = 4 replicates). *P* values were calculated using one-way ANOVA with Tukey’s post hoc test. **e**, Effect of 2.5 mM Glu in addition to 125 μM TTFA on viability after 72 h of culture in OCI-AML2 cells (*n* = 3 replicates). *P* values were calculated using two-way ANOVA with Tukey’s post hoc test. **f**, OCI-AML2 cell growth over 4 days in standard (2 mM, Gln^hi^) or reduced (0.1 mM, Gln^lo^) Gln with or without the addition of 1 mM DMS, DMKG or DMG (*n* = 3 replicates per time point, per group). *P* values were calculated using repeated-measures ANOVA with Tukey’s post hoc test. **g**, Effect of increasing concentrations of DMS, DMKG or DMG (0.5–2 mM) on viability in AML cells cultured in normal or reduced Gln for 4 days. Data points show the mean of *n* = 3 replicates per cell line. *P* values were calculated using two-way ANOVA with Tukey’s post hoc test. **h**,**i**, Effect of Glu loading with 5 mM DMG in combination with complex II inhibition (200 μM TTFA) or system *X*_*c*_ inhibition by 2.5 μM ERAS on viability (**h**) and relative intracellular Glu (**i**) levels (*n* = 3 replicates). *P* values were calculated using two-way ANOVA with Tukey’s post hoc test. **j**, Waterfall plot showing the effect of 5 mM DMG treatment on nucleotide metabolites in MOLM-13 AML cells after 18 h (fold changes (FCs) calculated from *n* = 4 replicates). For all experiments, data show the mean ± s.e.m. unless otherwise designated. XMP, xanthosine monophosphate; dATP, deoxyadenosine triphosphate; NMN, nicotinamide mononucleotide; NADP, nicotinamide adenine dinucleotide phosphate; GTP, guanosine triphosphate; SAH, S-adenosylhomocysteine; GDP, guanosine diphosphate; GDP-Man, GDP mannose; MTA, 5’-methylthioadenosine; AICAR, 5-amino-4-imidazolecarboxamide riboside; ADP, adenosine diphosphate. Panel **a** created with BioRender.com. Source Data

**Extended Data Fig. 6:**
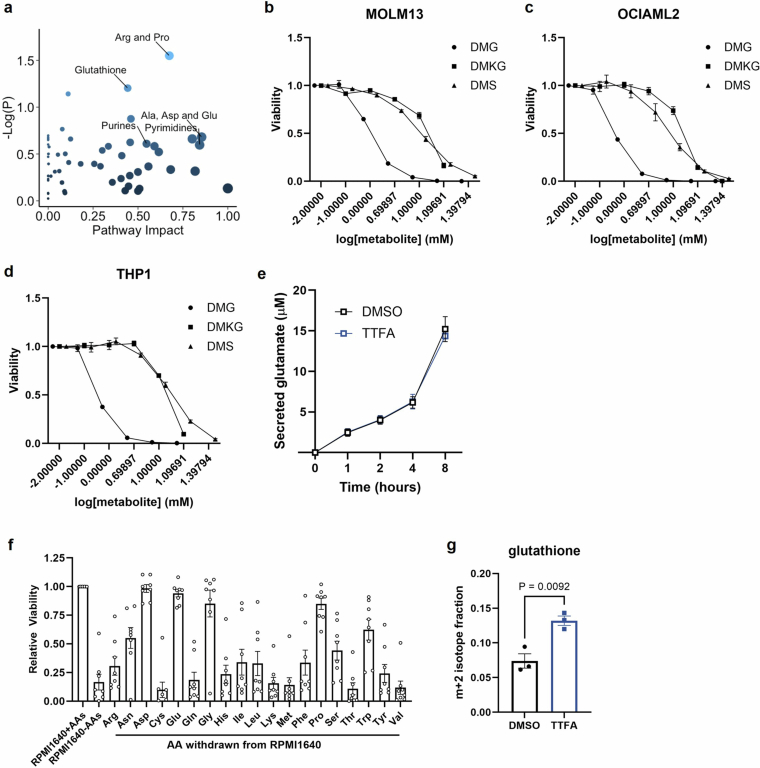
Consequences of perturbed metabolism in complex II-inhibited AML cells. **a**) Pathway Impact plot for steady state metabolites in MOLM-13 cells treated with DMSO or TTFA for 18 hr. Pathway Impact data and P values were calculated using Metaboanalyst software (Supplemental Table 3). **b-d**) Growth inhibition curves for (**b**) MOLM-13, (**c**) OCI-AML2 and (**d**) THP-1 cells cultured in the presence of increasing concentrations of DMG, DMKG or DMS. **e**) Glutamate secreted into glutamate-deficient media over 8 hr by OCI-AML2 cells cultured in DMSO or 200 μM TTFA. **f**) Viability of a panel of 8 AML cell lines cultured for 72 hr in amino-acid free RPMI1640 reconstituted with all amino acids (RPMI1640+AAs), devoid of all amino acids (RPMI1640-AAs), or with individual amino acid dropouts. Each point represents a cell line and is the average of n = 3 replicates for that cell line. **g**) Effect of Complex II inhibition on ^13^C_6_-glucose-derived carbon in glutathione (m + 2, from either glutamate or glycine). All data are shown as mean ± s.e.m. of n = 3 experiments unless otherwise specified. P value was calculated using an unpaired, two-tailed Student’s t-test. Source Data

Our isotope tracing studies showed that complex II blockade causes carbon accumulation in proximal metabolites succinate, α-KG and Glu. Furthermore, analysis of steady-state metabolites after TTFA treatment revealed specific effects on amino acid pathways including alanine, aspartate and Glu (Extended Data Fig. 6a and Supplementary Table 5). Therefore, we dissected the metabolic bottleneck upstream of complex II to determine the specific metabolite responsible for AML inhibition (Fig. 5a). To challenge AML cells with intracellular succinate, α-KG or Glu, we used cell-permeable dimethyl esters dimethyl-succinate (DMS), dimethyl-α-KG (DMKG) and dimethyl-Glu (DMG). Whereas supraphysiological doses of DMKG and DMS (up to 10 mM) were required to inhibit AML growth, DMG was tenfold more potent (Fig. 5b and Extended Data Fig. 6b–d). This selective sensitivity to DMG was seen across AML cells representing distinct genetic drivers (*KMT2A*, *FLT3*, *NRAS* and *TP53* mutated), supporting a broader metabolic sensitivity to Glu in AML.

We thus turned our attention to Glu. During purine synthesis, Glu is produced as Gln provides nitrogen to build the purine ring^25^. This occurs at two critical steps: when PRPP amidotransferase (PPAT) converts PRPP to phosphoribosyl-β amine, and again when phosphoribosylformylglycinamidine synthase (PFAS) converts formylglycinamide ribonucleotide to formylglycinamidine ribonucleotide (Fig. 3a). Using an orthogonal Glu luminescence assay, we confirmed that three structurally distinct complex II inhibitors increase intracellular Glu levels in multiple AML cell lines (Fig. 5c,d). At steady state, Glu was secreted by AML cells into Glu-deficient media (Extended Data Fig. 6e). Consequently, the addition of exogenous Glu to culture media induced a strong sensitization to complex II blockade (Fig. 5e).

TCA cycle anaplerosis is a key mechanism by which Gln supports cancer metabolism^26^. Consistent with this, withdrawal of Gln was among the most toxic individual amino acid dropouts for AML, and phenocopied the effects of complete amino acid starvation (Extended Data Fig. 6f). We tested the sufficiency of DMS, DMKG and DMG to rescue Gln withdrawal and loss of TCA anaplerosis. Remarkably, the addition of DMS and DMKG were able to restore AML growth in a dose-dependent manner, but in contrast DMG provided no benefit (Fig. 5f,g). These results corroborated our finding that Gln-derived carbon does not effectively enter the TCA cycle during complex II blockade (Fig. 4j–m) and provided direct evidence that DMG has secondary toxicity in AML that overrides its ability to restore TCA cycle anaplerosis.

These findings pointed to a cellular adaptation where AML cells must manage excess Glu during complex II inhibition to prevent toxic effects. Beyond providing carbon to the TCA cycle, Glu serves as a substrate for glutathione synthesis and regulates cystine import through system *X*_*c*_^27^ (Fig. 5a). Complex II inhibition substantially altered glutathione metabolism in AML cells, causing them to redirect carbon into glutathione synthesis (Extended Data Fig. 6g). Consistent with this concept, we found that the addition of erastin (ERAS), a system *X*_*c*_ inhibitor, further reduced cell viability and increased cellular Glu levels in the setting of combined treatment with TTFA and DMG (Fig. 5h,i). Thus, multiple strategies to increase intracellular Glu enhance the toxic effects of TTFA.

Our data support a model in which Glu accumulation upstream of complex II reduces AML growth and survival through purine synthesis inhibition. To test this hypothesis, we treated AML cells with DMG and performed nucleotide metabolomics. Consistently, we saw that DMG caused significant reductions in multiple purine metabolites including 5-amino-4-imidazolecarboxamide riboside, adenylosuccinate, adenosine diphosphate and ATP (Fig. 5j and Supplementary Table 6). These results reveal a metabolic vulnerability in AML where complex II inhibition triggers Glu accumulation, which suppresses purine synthesis and enhances cell death. This mechanism suggests potential therapeutic strategies combining complex II inhibition with approaches that increase cellular Glu.

## Glu broadly impacts the AML proteome

**Fig. 6:**
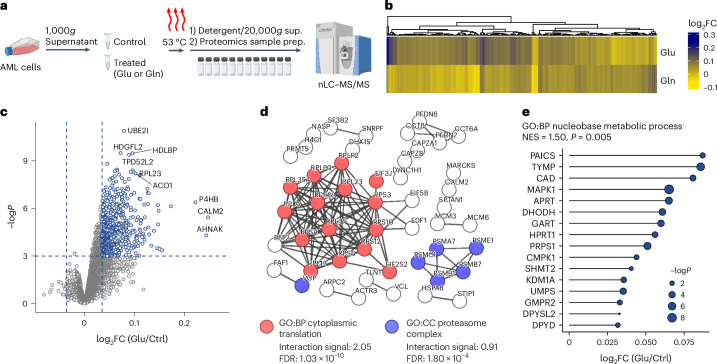
Glu interacts widely within the AML proteome. **a**, Overview of isothermal proteome profiling method to detect protein–metabolite interactions of Glu or Gln in AML cells. **b**, Heat map showing the log_2_FC in abundance after Glu or Gln binding for *n* = 287 enriched AML proteins (*z*-score ≥ 2 or ≤ −2 and *P* value ≤ 0.05 for either Glu or Gln versus control, based on limma analysis). **c**, Volcano plot showing the top Glu-interacting proteins relative to control. *P* values were generated using limma. **d**, STRING-generated protein–protein interaction network for Glu-interacting proteins with *z-*score ≥ 2 and *P* value ≤ 0.05. Edges show physical interactions with highest interaction strength (≥0.9) and disconnected nodes are not shown. Two representative pathways, GO:BP ‘cytoplasmic translation’ and GO:CC ‘proteasome complex’, are shown with pathway components highlighted in respective colours and STRING interaction score listed. **e**, Lollipop plot showing the log_2_FC and enrichment for the proteins making up the nucleobase metabolic process GO category. *P* values were generated between control and Glu-treated cells using limma. NES, normalized enrichment score. Panel **a** created with BioRender.com. Source Data

**Extended Data Fig. 7:**
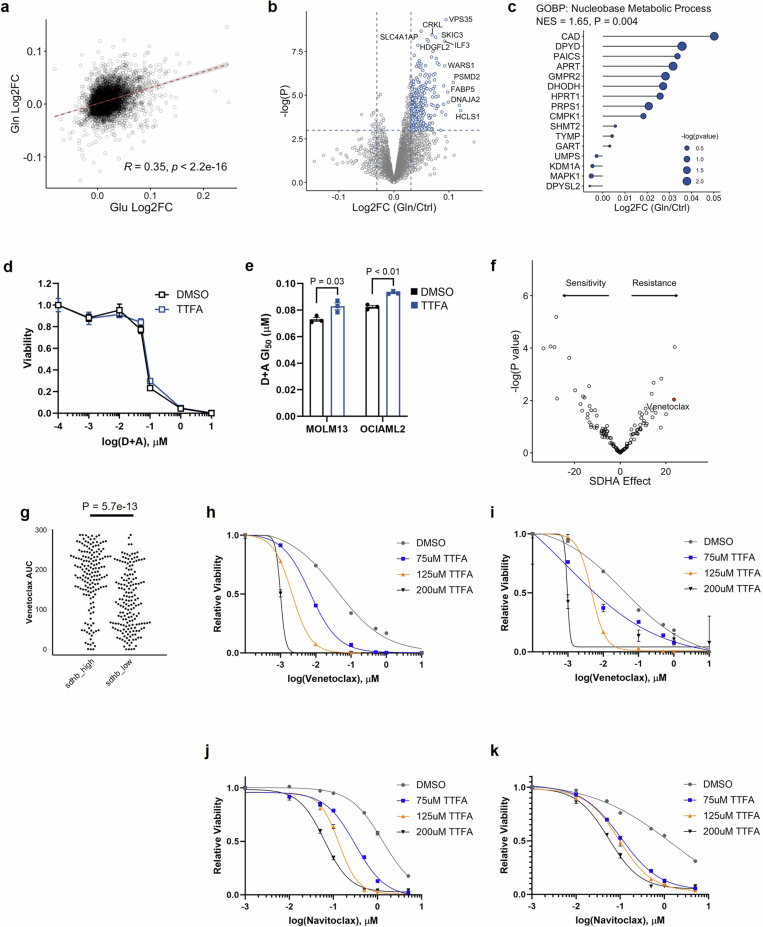
Profiling glutamate or glutamine protein interactions and complex II-dependent drug sensitivity in AML cells. **a**) Correlation plot showing log-2 fold changes in AML protein abundance after glutamate or glutamine treatment. P-value was generated using a two-tailed t-test based on the Pearson correlation coefficient. **b**) Volcano plot of top interacting proteins with glutamine in AML cells. P-values were generated between control and Gln-treatment using limma. **c**) Pathway enrichment analysis showing Gene Ontology Biologic Process (GOBP) “Nucleobase Metabolic Process” pathway components and the effect of glutamine treatment on protein abundance. P-values were generated between control and Gln-treatment using limma. **d**) Relative viability of OCI-AML2 cells treated with increasing concentrations of daunorubicin and cytarabine (D + A) in the absence or presence of 50 μM TTFA. The D + A concentration shown is the sum (concentration of each drug is 50% of value shown). **e**) Effect of TTFA on the GI_50_ values for D + A chemotherapy in MOLM-13 and OCI-AML2 cells. **f**) Effect of *SDHA* gene expression on drug sensitivity in the Beat AML cohort. **g**) Beat AML area under the curve (AUC) data for venetoclax treatment, stratified by *SDHB* levels above (sdhb_high) or below (sdhb_low) the median. Each dot represents a patient sample from the study. **h-i**) Effect of Complex II inhibition with increasing doses of TTFA on AML sensitivity to venetoclax in (**h**) MOLM-13 or (**i**) OCI-AML2 cells. **j-k**) Effect of Complex II inhibition with increasing doses of TTFA on AML sensitivity to navitoclax in (**j**) MOLM-13 and (**k**) OCI-AML2 cells. GI_50_ values for venetoclax or navitoclax were calculated from n = 3 experimental replicates and presented in Fig. 7h–i. Data shown are mean ± s.e.m. P values in (**e**) and (**g**) were calculated using unpaired, two-tailed Student’s t-tests. Source Data

To begin to understand how Glu reduces purines, we performed isothermal proteome profiling to assess protein–metabolite interactions in AML (Fig. 6a). We identified over 5,000 proteins in AML lysates (Supplementary Tables 7 and 8), consistent with the proteome coverage reported in prior AML drug–protein interaction screens^28^. This included 287 significantly interacting proteins for either Glu or Gln, 199 of which were shared by both metabolites (Fig. 6b and Extended Data Fig. 7a). The top Glu and Gln interactions included proteins involved in metabolism and protein dynamics (Fig. 6c and Extended Data Fig. 7b), suggesting important effects in AML. In support of this, physical protein clusters involving translation and proteasome function were both enriched with high confidence in Glu-interacting proteins (Fig. 6d). A pathway enrichment analysis identified the GO:BP ‘nucleobase metabolic process’, which includes de novo purine biosynthesis enzymes PAICS and GART, as significantly enriched for both Glu and Gln binding (Fig. 6e and Extended Data Fig. 7c). Overall, these data corroborate the notion that Glu accumulation may directly impact purine biosynthesis while defining the broader members of the proteome engaged by this metabolite, suggesting that it may also drive other pleiotropic effects.

## Elevated complex II expression is a feature of high-risk human AML and promotes resistance to targeted therapy

**Fig. 7:**
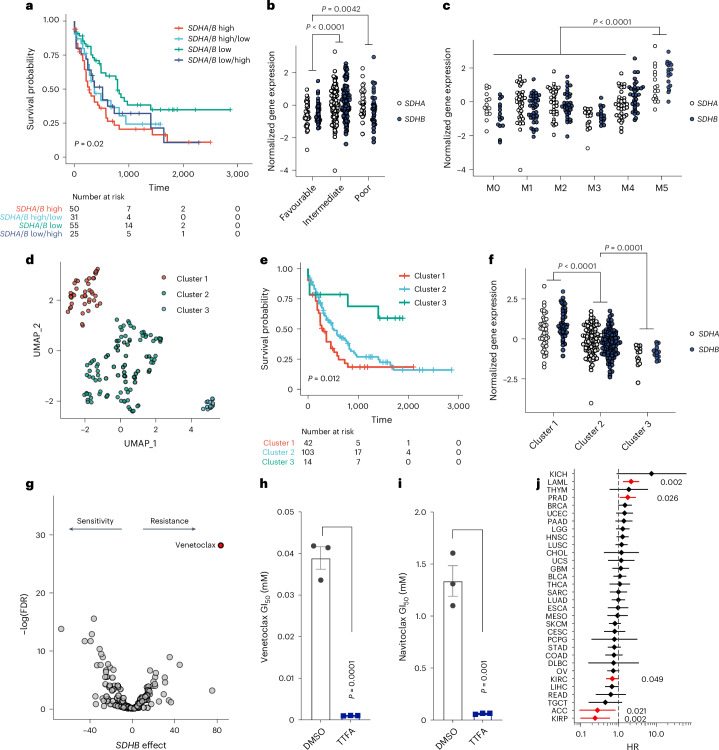
Complex II genes are overexpressed in high-risk AML resistant to BCL-2 inhibitors. **a**, Kaplan–Meier curve showing overall survival of the TCGA-LAML cohort, stratified by *SDHA* or *SDHB* expression above or below the mean. **b**, *SDHA* and *SDHB* gene expression (normalized to *z*-score) in the TCGA cohort, stratified by AML risk status. **c**, *SDHA* and *SDHB* gene expression in the TCGA cohort, grouped by morphological AML subclass. Data in **b** and **c** are for *n* = 161 samples from the TCGA-LAML cohort. **d**, UMAP analysis of TCGA-LAML data, showing three clusters based on whole transcriptome data. **e**, Overall survival for AML stratified by UMAP cluster. **f**, *SDHA* and *SDHB* expression stratified by TCGA UMAP cluster, with the highest expression in clusters with poorer outcomes. Data in **d** and **e** are for *n* = 159 samples with full transcriptome gene counts in the TCGA-LAML cohort. **g**, Volcano plot showing the association between *SDHB* gene expression and small-molecule inhibitor sensitivity in the Beat AML cohort^29^. Higher *SDHB* expression in human blasts was associated with resistance to inhibitors on the right of the plot and sensitivity to inhibitors on the left. **h**,**i**, Effect of complex II inhibition (200 μM TTFA) on the GI_50_ of MOLM-13 AML cells to the BCL-2 family inhibitors venetoclax (**h**) and navitoclax (**i**). Data shown are the mean ± s.e.m. for *n* = 3 replicates. **j**, HR plot showing the risk of death associated with elevated *SDHA* and *SDHB* expression across all TCGA cancer types. The cohort for each cancer type was divided above or below the mean for both *SDHA* and *SDHB* expression levels as in **a**, with *SDHA*-high/*SDHB*-low and *SDHA*-low/*SDHB*-high groups excluded. Data show the mean HR (diamond) with 95% confidence interval (line segment). Red indicates cancer types with significant HRs, with *P* values shown at the side. *P* values were calculated using a log-rank test (**a**,**e**); one-way ANOVA and Tukey’s post hoc test (**b**,**c**,**f**); an unpaired, two-tailed Student’s *t*-test (**h**,**i**); or Cox proportional hazard analysis (**j**). Source Data

Our metabolic studies revealed that complex II has critical effects on Glu metabolism and supports AML growth through purine biosynthesis regulation. These findings prompted us to investigate complex II’s role in human AML. Survival analysis of The Cancer Genome Atlas (TCGA) data revealed that high expression of both complex II subunits *SDHA* and *SDHB* was associated with the poorest survival for individuals with AML (Fig. 7a). *SDHA* and *SDHB* expression levels were higher in adverse risk groups and in AML with monocytic (M5) morphology (Fig. 7b,c). Extending this approach, we performed a dimension reduction analysis on TCGA-LAML transcriptomes using uniform manifold approximation and projection (UMAP) and found three distinct clusters (Fig. 7d), again with the poorest-surviving group showing the highest *SDHA* and *SDHB* expression (Fig. 7e,f). These data identify complex II as a high-risk molecular feature in human AML.

Next, we investigated the effects of complex II on treatment response. Despite *SDHA* and *SDHB* overexpression in high-risk disease, complex II inhibition did not sensitize AML cells to conventional chemotherapy with cytarabine and daunorubicin (Extended Data Fig. 7d,e). To better understand the effects of complex II, we turned to the Beat AML dataset^29^. We performed a regression analysis to identify the association between drug sensitivity and complex II gene expression. We found high expression of both *SDHA* and *SDHB* correlated with resistance to targeted therapies. Most notably, high *SDHB* expression was associated with resistance to the BCL-2 inhibitor venetoclax (Fig. 7g). High *SDHA* expression was also associated with venetoclax resistance to a lesser extent (Extended Data Fig. 7f). This suggested a therapeutic opportunity by combining complex II and BCL-2 inhibition. Indeed, TTFA treatment sensitized AML cells to both venetoclax and the related BCL-2/BCL-xL inhibitor navitoclax (Fig. 7h,i and Extended Data Fig. 7g–k).

Complex II’s role in AML stands out among human cancers. Calculating the hazard ratio (HR) of high combined *SDHA* and *SDHB* expression across 33 cancer types in TCGA, we found significantly increased mortality risk in only two cancers, with AML being one (Fig. 7j). This contrasts sharply with tumours like adrenocortical carcinoma, and renal papillary carcinoma, where high *SDHA* and *SDHB* expression associated with improved survival. In certain cancers, complex II acts as a tumour suppressor and its loss drives hereditary cancer predisposition syndromes^30,31^. This opposite effect, where complex II promotes disease in AML but suppresses it in other cancers, highlights its unique role in AML progression and suggests targeted therapeutic opportunities.

## Discussion

We developed an algorithm that moves beyond established gene–gene coessentiality mapping to reveal complex interactions across biological pathways. We applied this approach to examine relationships among the protein complexes of the ETC and discovered a new association between complex II and purine synthesis. Furthermore, we demonstrate that complex II directly regulates purine synthesis to maintain cell proliferation. Mechanistically, we provide evidence that an important function of complex II is to metabolize carbon from Gln. Following complex II inhibition, Gln carbon (that is, Glu) processing through the TCA cycle is reduced, which directly reduces purines.

Our finding that complex II regulates purine synthesis adds to a growing body of work linking the ETC and TCA cycle to non-canonical functions, particularly nucleotide synthesis. Landmark studies revealed that the major role of mitochondrial complex I in maintaining proliferation is not through ATP production but rather through NAD^+^/NADH-dependent aspartate biosynthesis^5,6^. The reduction in NAD^+^/NADH also results in TCA cycle inhibition and depletion of asparagine-mediated nucleotide production^7^. Recently, a number of studies have connected the ETC and TCA cycle enzymes directly to purine synthesis, including complex I^8^, OGDH^32^ and fumarate hydratase^33^. Models of fumarate hydratase deficiency were shown to block purine synthesis via fumarate accumulation driving the reversal of adenylosuccinate lyase (ADSL)^33^. This observation supports our finding that Glu accumulation upstream of complex II depletes purines, suggesting a new mechanism of cellular sensing. Additional work is needed to determine the specific mechanism by which Glu exerts these effects.

Our work also highlights the potential for complex II to be used therapeutically against cancer. Directly targeting OxPhos for cancer therapy has proven to be challenging, mainly due to a lack of therapeutic window and dose-limiting toxicities that are unable to achieve clinical benefit. In particular, complex I inhibition has recently been associated with neurotoxicities^34^. However, some evidence suggests that complex II may be a viable therapeutic target.

First, we find a subset of cancer cell lines are exquisitely dependent on complex II, suggesting there may be an exploitable therapeutic window in specific cancers. Our data suggest this may be the case for AML in particular, where OxPhos inhibition could be combined with venetoclax and azacitidine—agents well known to selectively eradicate leukaemic stem cells^35^. We show that complex II inhibition sensitizes AML cells to BCL-2 inhibition, providing a possible area of exploration in venetoclax-resistant disease^36^. In addition to AML, our data also point to a broader essential role for complex II in haematolymphoid malignancies, including B cell acute lymphoblastic leukaemia and certain lymphomas, particularly anaplastic large cell lymphoma and DLBCL. These findings highlight the need for further studies on whether the essential functions of complex II are shared among subsets of cancers. In fact, using TCGA data, we show that the increased risk of mortality with elevated complex II gene expression is not a pan-cancer phenomenon, further implicating complex II in AML and distinguishing it from tumours in which complex II acts as a tumour suppressor.

Second, several studies have shown that complex II (*Sdhd*) ablation is tolerable in adult mice^37,38^. Models of partial or heterozygous whole-body knockout of *Sdhd* and *Sdhc* in adult mice reduced SDH activity and were well tolerated, with no major pathophysiology observed other than reduced carotid body volume^37,38^. Of note, germline mutations in *SDH* subunits predispose humans to a small subset of cancers, including pheochromocytoma, paraganglioma, gastrointestinal stromal and renal tumours, which are manifested after loss of the wild-type allele^39^. However, *Sdh* loss in mice was not associated with tumour development^37,38^. Using TCGA data, we have found that the overall risk of elevated *SDHA* and *SDHB* expression is cancer dependent, suggesting that complex II targeting may be more tissue selective and may broaden the therapeutic window. Additionally, mitochondrial-targeted lonidamine, a clinically safe glycolysis inhibitor found to inhibit complex II, was well tolerated in mice and inhibited the growth of lung cancer xenografts^40^. However, other pharmacological inhibitors of complex II, 3-NP and malonate, have been investigated in vivo and found to cause striatal defects analogous to those in Huntington’s disease with long-term use^41,42^. Since neurotoxicity is a primary concern with ETC inhibition, this raises the possibility of creating a brain-impermeant complex II inhibitor to retain its anti-tumorigenic properties while avoiding serious side effects.

Finally, we note that *OGDH* ablation was reported to have similar inhibitory effects on purine synthesis, but ascribed to different mechanisms^43^. Because knockdown of *Ogdh* in mice results in mild side effects but is generally well tolerated^43^, it may be an alternative therapeutically targetable node in complex II-dependent cancers. Overall, these data support a key role of the TCA cycle and ETC in supporting purine biosynthesis in AML and other haematolymphoid malignancies.

## Methods

### Cell lines and reagents

All cell lines were purchased from the American Type Culture Collection or Duke University Life Science Facility and maintained at 37 °C with 5% CO_2_. All cell lines were cultured in RPMI 1640 medium with 10% FBS and 1% penicillin–streptomycin. Amino acid-deficient RPMI 1640 was purchased from US Biological. For experiments with addition of exogenous metabolites and amino acid/Glu modulation experiments, RPMI 1640 medium with 10% dialysed FBS and 1% penicillin–streptomycin was used. For the CRISPR screen, DMEM high-glucose medium was used. TTFA and AA5 were purchased from Cayman Chemical. LTX, 3-NP and all exogenous metabolites were purchased from Sigma-Aldrich, except for cytosine and thymine, which were purchased from TargetMol. Brequinar was purchased from MedChemExpress. ERAS was purchased from Apexbio. DMS was purchased from Thermo Fisher. DMKG and DMG were purchased from Sigma. Venetoclax and navitoclax were purchased from SelleckChem.

### Pathway embedding and CCA

To develop a pathway–pathway coessentiality map, we started with essentiality data from the Cancer Dependency Map. Gene sets representing pathways were identified using the MSigDB resource^13^. Next, principal component analysis (PCA) was performed independently for each pathway. The first four PCs for each pathway were selected, reducing all pathways from their original respective sizes to a common size of four components. This dimension reduction was performed to make the pathways comparable and reduce overfitting of the CCA model, which can occur when the number of variables is high and large differences exist between the number of variables in compared gene sets. Finally, pathway coessentiality data were extracted by computing CCA on the first four PCs and selecting the first canonical correlation (CC1). CC1 values were used to represent pathway coessentiality with a range of 0–1.

For ETC embeddings, custom pathways were created using core and accessory ETC gene subunits. Genes for each complex were selected as annotated in Mitocarta 3.0. PCA, extraction of top PCs and CC1 calculation were performed identically to the pathway analysis described above.

To determine how PC extraction and CCA changed correlation scores relative to simple coessentiality correlations, we extracted dependency scores for OxPhos genes from DepMap and used them to calculate coessentiality between each gene and each GO pathway. These single gene–pathway correlations were combined and displayed as ‘gene–pathway’ values corresponding to their associated ETC complex. To determine the effect of PC extraction and CCA, these calculated gene–pathway values were compared to CC1 scores that were calculated using PC extraction and CCA for the same genes. These values are represented as ‘complex-pathway’. The top pathways with unique complex II associations were selected by filtering for pathways with CC1 values ≥ 1 standard deviation above the median for complex II and ≤1 standard deviation above the median for other ETC complexes (Supplementary Table 2).

To compare complex II dependency scores between cancer sublineages, genes were grouped by ETC complex. The mean dependency score was calculated within a lineage or sublineage, as well as the 5%, 50% and 95% intervals. Differences in ETC complex dependency between sublineages was calculated using a two-tailed Student’s *t*-test between each lineage and the aggregate of cells not in the lineage.

### Cellular viability assays

Cells were seeded at an equal density ranging from 2 to 10 × 10^3^ cells per cm^2^ in tissue culture plates and allowed to equilibrate. After 24 h, treatment drugs or metabolites were added to cells in triplicate. Cell viability was measured 48–96 h later using Cell-Titer Glo (Promega) at a 1:1 vol/vol ratio on a Tecan plate reader (Infinite M1000 PRO). The drug and metabolite concentrations, as well as the specific timing of each experiment, are noted in the figure legends. Relative cell viability was calculated by normalizing treatment luminescence values to vehicle-treated wells. For experiments with drug or with metabolite combinations, the second drug or metabolite was kept at a constant concentration across all wells and the first drug was applied on top of the background drug or metabolite(s). Relative cell viability was then normalized to the luminescence of cells in the presence of the background drug or metabolite alone.

### MLL-AF9 mouse model

Experiments involving mice were conducted in accordance with approved animal use and care protocols under the French National Ethics Committee on Animal Care (authorization number APAFIS 8909-2017021413452743 v1). Equal numbers of male and female mice were maintained on a 12–12-h light–dark cycle with ad libitum access to food (standard chow) and water. For experiments examining the effect of complex II in AML in vivo, granulocyte–macrophage progenitor cells expressing dsRed in tandem with the MLL-AF9 leukaemia translocation were isolated and transfected with lentivirus constitutively expressing Venus YFP and a doxycycline-inducible Crimson far-red RFP in tandem with either non-targeting shRNA (shNT) or one of two independent shRNA hairpins targeting complex II subunit *Sdhb* (sh*Sdhb*.1 and sh*Sdhb*.2, respectively). The effect of doxycycline on *Sdhb* expression in shNT, sh*Sdhb*.1 or sh*Sdhb*.2 cells was determined in transfected MLL-AF9 cells after 3 days.

Transfected MLL-AF9 cells were transplanted via tail vein injection into syngeneic recipients that were randomized to receive shNT, sh*Sdhb*.1 or sh*Sdhb*.2 transfected cells. Leukaemia burden was quantified as the proportion of AML cells expressing the inducible transcript (Crimson^+^Venus^+^) relative to total AML cells (Venus^+^) in bone marrow or spleen. For survival studies, MLL-AF9 transplanted mice were followed after engraftment until they displayed signs of overt leukaemia or if moribund as described by the ethics committee and institutional review board documentation.

### Genome-wide MinLib CRISPR–Cas9 drug sensitizer screen and analysis

OCI-AML2 cells were transduced with Cas9 and selected for 5 days with blasticidin. OCI-AML2-Cas9 cells were seeded at a density of 2.5 × 10^6^ cells per well in six-well plates in DMEM high-glucose media and spinfected with MinLib library virus and polybrene at a multiplicity of infection of 0.3. Media were replaced with fresh media containing puromycin 24 h later; selection occurred for 3 days. Cells were then split into two conditions, DMSO or TTFA (50 µM) in triplicate, and routinely passaged for 3 weeks. A minimum coverage of 500× was maintained at all times during the screen. Cell pellets of at least 500× coverage (20 × 10^6^ cells) were obtained immediately after completion of selection (*T*_0_), at every passage including the halfway point (*T*_mid_), and following completion of the screen (*T*_F_). Genomic DNA from pellets were extracted using the QIAamp Blood Maxi Kit (Qiagen). The single guide RNAs (sgRNAs) from each sample were PCR amplified from genomic DNA using NEBNext Ultra II Q5 Master Mix (NEB). Another PCR added unique combinations of staggered forward and reverse primers with barcodes to amplified sgRNAs for deconvolution. The PCR2 product was purified to yield barcoded sgRNAs using SPRIselect beads (Beckman Coulter) and right-sided selection, which were quantified using the Quant-iT dsDNA Broad Range Assay Kit (Thermo Fisher). Samples were pooled evenly and sequenced on an Illumina NextSeq 500 with 75-base pair single-end sequencing. sgRNA counts were analysed using Models-based Analysis of Genome-wide CRISPR–Cas9 Knockout (MAGeCK) software using three time points: *T*_0_, *T*_mid_ and *T*_F_. Beta scores comparing TTFA to DMSO over time linearly were generated with MAGeCK^44^. *n* = 1,491 genes from DepMap that were inferred as Common Essential genes in cancer were removed from the analysis. One purine biosynthesis gene, *PPAT*, was considered a common essential cancer gene but was retained in the analysis for completeness.

### Measurement of ATP levels

Cells were seeded at a density of 10 × 10^3^ cells per well in a 96-well plate. After 24 h, cells were treated with DMSO or TTFA (200 μM) for 30 min in triplicate, before viability was impacted, and assayed using Cell-Titer Glo (Promega) on a Tecan plate reader (Infinite M1000 PRO). Luminescence values were normalized to DMSO-only conditions to obtain relative ATP levels.

### Metabolite profiling and stable-isotope tracing

For steady-state metabolite profiling, 1 × 10^6^ cells were seeded in 10-cm plates. After 24 h, DMSO or TTFA (200 μM) was added to cells in triplicate. After 18 h of treatment, cells were harvested by centrifugation, washed with ice-cold 0.9% saline twice, and 80% (vol/vol) methanol pre-chilled to −80 °C was added to cells on dry ice to quench and extract metabolites. Lysates were incubated at −80 °C for 5 min, brought to room temperature for 15 min and then vortexed for 1 min. The freeze–thaw steps were repeated three times, after which samples were incubated overnight at −20 °C to precipitate proteins. Samples were vortexed for 30 s and centrifuged for 15 min at 20,000*g* at 4 °C. Supernatants were stored at −80 °C until analysis by LC–MS. Samples were normalized to the protein concentration contained in the cell pellet.

For stable-isotope tracing experiments, 1 × 10^6^ cells were seeded in 10-cm plates. After 24 h, cell culture medium was changed to glucose-free or Gln-free medium containing 10% dialysed FBS and 11.1 mM ^13^C_6_-glucose, 2 mM ^15^N_1_-Gln-amide or ^13^C_5_-Gln (Cambridge Isotope Laboratories), respectively. Simultaneously, vehicle, TTFA (200 μM) or LTX (150 nM) was added for 24 h. Metabolite extraction was performed as above. Samples were normalized to cell count.

For quantitative nucleotide metabolomics experiments, 30 × 10^6^ cells were seeded in 15-cm plates with 10% dialysed FBS as above. After 24 h, cells were treated with 5 mM DMG for an additional 18 h. Cells were harvested into pre-chilled 100% methanol and flash frozen in liquid nitrogen for subsequent metabolite extraction.

For all metabolomics experiments, samples were reconstituted after extraction by first drying the extraction solution using a SpeedVac and 60% acetonitrile was added to the tube, followed by vortexing for 30 s. Reconstituted samples were then centrifuged for 30 min at 20,000*g* at 4 °C. Supernatant was collected for LC–MS analysis.

Samples were analysed by high-performance liquid chromatography and high-resolution mass spectrometry and tandem mass spectrometry (HPLC–MS/MS). Specifically, the system consisted of a Thermo Q Exactive in line with an electrospray source and an UltiMate 3000 (Thermo) series HPLC consisting of a binary pump, degasser and autosampler outfitted with an Xbridge Amide column (Waters; dimensions of 3.0 mm × 100 mm and a 3.5-µm particle size). The mobile phase A contained 95% (vol/vol) water, 5% (vol/vol) acetonitrile, 10 mM ammonium hydroxide, 10 mM ammonium acetate, pH 9.0; B was 100% acetonitrile. The gradient was as follows: 0 min, 15% A; 2.5 min, 30% A; 7 min, 43% A; 16 min, 62% A; 16.1–18 min, 75% A; 18–25 min, 15% A with a flow rate of 150 μl min^−1^. The capillary of the electrospray ionization source was set to 275 °C, with sheath gas at 35 arbitrary units, auxiliary gas at 5 arbitrary units and the spray voltage at 4.0 kV. In positive/negative polarity switching mode, an *m*/*z* scan range from 60 to 900 was chosen and MS1 data were collected at a resolution of 70,000. The automatic gain control (AGC) target was set at 1 × 10^6^ and the maximum injection time (IT) was 200 ms. The top five precursor ions were subsequently fragmented, in a data-dependent manner, using the higher energy collisional dissociation cell set to 30% normalized collision energy in MS2 at a resolution power of 17,500. Besides matching *m/z*, metabolites are identified by matching either retention time with analytical standards and/or MS2 fragmentation pattern. Data acquisition and analysis were carried out by Xcalibur 4.1 software and TraceFinder 4.1 software, respectively (both from Thermo Fisher Scientific). For isotope tracing experiments, natural isotope abundance was corrected for using AccuCor^45^. For steady-state and quantitative nucleotide metabolomics experiments, data were normalized using log transformation and Pareto scaling to determine fold changes between control and experimental conditions.

### Glu measurements

Cells were seeded at 0.5–1 × 10^5^ per cm^2^ in culture plates. After 24 h, DMSO or a given drug or a metabolite was added to cells in triplicate for an additional 24–48 h. At the end of the culture period, an aliquot of cells from the cultured plates was used to calculate viability with the Cell-Titer Glo assay. A sample of the remaining cells (representing 20–50 × 10^3^ input cells, to be within linear range of the luminescence signal) were harvested by centrifugation and washed twice with ice-cold PBS. To lyse cells and inactivate metabolism, 0.6 N HCl was added for 5 min. An equivalent volume of 1 mM Tris Base was then added to neutralize lysates. The Glutamate-Glo assay (Promega) was performed according to the manufacturer’s instructions to determine Glu levels. A standard curve up to 50 μM Glu was performed with each experiment. Glu levels were normalized to cell viability for each sample to correct for differences in input cell number and then normalized to vehicle-only samples to obtain relative Glu levels.

### Isothermal proteome profiling

AML extracts were prepared from a flash frozen-cell pellet consisting of MOLM-13, OCI-AML2, MV-4-11 and THP-1 AML cells. The pellet was thawed in ice-cold PBS lysis buffer containing protease and phosphatase inhibitors, as well as 2 mM TCEP (Sigma-Aldrich) and manually homogenized with a glass homogenizer on ice. Protein was collected from the supernatant with two sequential 1000*g* spins.

#### Isothermal shift assay

In each experiment, an *n* = 3 replicates for each test group were performed in duplicate. Solutions of L-Gln and L-glutamic acid (Sigma-Aldrich) were made in PBS (pH 7.4), and the final treatment concentration of amino acid used was 0.5 mM. The assay was initiated by the addition of AML extract to a lysis buffer/metabolite solution and the binding step was performed at 22 °C for 3 min. A thermocycler (Biometra) was used for thermal challenge at 53 °C for 3 min, and then cooled to 4 °C for 3 min. The assay mixture was then treated with 0.8% NP-40 and kept in a 4 °C cold room on a tube rotator. Corresponding duplicate tubes were pooled across the *n* = 3 replicates, and the soluble protein fraction was isolated by centrifugation at 20,000*g* for 20 min at 4 °C. Aliquots of supernatant were collected for protein determination (BCA assay) or proteomics sample preparation. For protein digestion, an equal volume of the 20,000*g* supernatant of each sample at 22 °C was spiked with 2 µl (0.46 µg) of an intact protein standard mix of six proteins (Thermo Fisher Scientific). Subsequently, an equal volume of 2× SDS Protein Solubilization Buffer (100 mM TEAB, pH 8.5, 10% SDS) was added and mixed by vortexing. The protein mix was reduced using 5 mM dithiothreitol at 55 °C for 15 min. After cooling to room temperature for 30 min, the samples were alkylated by adding 15 mM (final concentration) iodoacetamide and incubating for 30 min at room temperature in the dark. Finally, the iodoacetamide was quenched by adding dithiothreitol to a final concentration of 15 mM and incubating for 15 min at room temperature in the dark. The solution was acidified by adding phosphoric acid to a final concentration of 2.5%. To the acidified solution, a volume of S-Trap Binding/Wash Buffer (90% methanol, 100 mM TEAB, pH 7.55) was added that was six times the accumulated volume of the previous steps. S-Trap micro columns (Protifi) attached to a vacuum manifold were applied to pull sample through the column and then washed four times with 150 µl S-Trap Binding/Wash Buffer. Trypsin stock solution (Promega) was diluted at 0.05 µg µl^−1^ in digestion buffer (50 mM TEAB, pH 8.5) and 20 µl was added to each column. Each column was placed into a microcentrifuge tube and incubated in a non-shaking covered water bath at 47 °C for 1 h. Following digestion, the resulting peptides were eluted by the following three steps: (1) added 40 µl 50 mM TEAB, pH 8.5 and centrifuged at 4,000g, 2 min, at 22 °C, into a fresh tube; (2) added 40 µl 0.2% formic acid and repeated the centrifugation into the same tube; (3) added 40 µl 50% acetonitrile and repeated the centrifugation into the same tube. Peptides were dried in a SpeedVac with no heat overnight and then stored at −80 °C.

#### TMT labelling

Nine tags from a 10-plex tandem mass tag (TMT) kit (0.2 mg per tag; Thermo Fisher Scientific) were used for labelling peptides. To each peptide sample, 10 µl of 200 mM TEAB (Thermo Fisher Scientific) was added. Tags were centrifuged for 1 min at 14,000 rpm and then resuspended in 12.5 µl 100% anhydrous acetonitrile (Sigma-Aldrich). To each peptide sample, 5 µl of one tag was added, vortexed and pulse centrifuged. Samples were incubated for 4 h at 22 °C in a Thermomixer at 1,000 rpm. The reaction was quenched by adding 0.8 µl of 5.25% hydroxylamine, vortexed and incubated for 15 min at 22 °C in a Thermomixer at 1,000 rpm. After the incubation, samples were centrifuged for 1 min at 14,000 rpm at room temperature. All samples within the 9-plex were pooled, vortexed, pulse spun and frozen on dry ice. The sample was dried in a SpeedVac with no heat overnight and then stored at −80 °C. TMT-labelled peptides were further purified by SPE using a Sep-Pak 50-mg column, with peptides eluted once with 0.5 ml of 25% acetonitrile/0.1% TFA and twice with 0.5 ml 50% acetonitrile/0.1% TFA. The eluate was frozen and dried overnight in a SpeedVac with no heat and stored at −80 °C.

#### Peptide fractionation

The peptide sample mixture was reconstituted in 225 μl of 50 mM ammonium bicarbonate, and 100 μl was fractionated by high pH reversed-phase chromatography into 12 HPLC fractions using the concatenation strategy (60 initial eluate collections of 30 s each, pooling every 12th fraction)^46^. The eluate was frozen on dry ice, dried in a SpeedVac with no heat, resuspended in 16.7 μl (targeting ~0.5 μg μl^−1^ in nine-sample mixes) of 0.1% formic acid and transferred to LC–MS autosampler vials.

#### Proteomic data acquisition

Quantitative nanoLC–MS/MS (nLC–MS/MS) analyses were performed on a Q Exactive Plus Orbitrap mass spectrometer coupled to an EASY-nLC UPLC system via a nanoelectrospray ionization source (all from Thermo Fisher Scientific). Before analysis of experimental samples, the instrument was calibrated and nLC–MS/MS performance validated using a peptide standard (cytochrome c digest). The 12 fractions for each TMT kit were each run twice, with 4 μl injected for each. For each run, peptides were first trapped (500 bar max pressure) on an Acclaim PepMap (Thermo) 100 trapping column (3 μm, 75 μm × 20 mm) and separated on an Acclaim PepMap (Thermo) RSLC C18 analytical column (2 μm 100 C18, 75 μm × 500 mm column) over a 105-min gradient of solvent A (0.1% formic acid) to solvent B (90% acetonitrile/0.1% formic acid) at 300 nl min^−1^ and a column temperature of 55 °C. MS1 spectra were collected at a resolution (*r*) of 70,000, a target AGC value of 3 × 10^6^ ions, and a maximum IT of 60 ms, and MS2 spectra (*r* = 35,000, AGC = 1 × 10^5^, max IT = 60 ms) were collected by data-dependent acquisition using a loop count of 10, an isolation window (IW) of 0.7 *m/z*, normalized collision energy of 30 and dynamic exclusion enabled for 30 s.

#### Proteomic data analysis

Raw data files were processed in Proteome Discoverer 3.1 (PD3.1, Thermo Fisher Scientific), with all fractions from a given TMT kit searched together. After MS1 precursor mass recalibration was performed via a pre-search using the ‘spectrum files RC’ node, data were re-searched with Sequest HT against UniProt human (UP000005640, downloaded 02 March 2025) complete proteome database and corresponding ‘decoy’ reversed protein sequences. All runs considered TMT10plex (229.163 kDa on peptide N terminus and K) and carbamidomethyl (57.021 kDa on C) as fixed modifications, and oxidation (15.995 kDa on M) as variable, with up to two missed cleavages (full trypsin specificity). After re-scoring with Inferys, peptide-spectrum match scores were filtered to a 1% false discovery rate (FDR) using Percolator, collapsing peptide-spectrum match scores from both experiments to unique peptides with Peptide Validator while maintaining a 1% peptide-level FDR. Peptides were grouped to proteins (strict parsimony), which were filtered to 1% FDR with Protein FDR Validator. Quantification was normalized for the measured abundance of the six protein standards to adjust for any subtle differences introduced during the digestion, TMT labelling and solid phase extraction steps. ‘Master proteins’, or representatives of a group of proteins containing the same peptides, were used for quantitative comparison (using quantitative values from unique peptides), with imputation performed by low abundance resampling. Proteins were normalized to the total abundance of all proteins within each sample to account for differences in overall protein concentration between samples. Following normalization, log transformation and Pareto scaling were applied to each protein’s abundance data. Differential protein abundance analysis was performed using the limma package.

### TCGA cancer survival data analysis

TCGA survival and gene expression data were collected using the TCGA survival tool (https://www.tcga-survival.com/). For survival analysis by gene expression status, cohorts were divided into ‘gene-high’ and ‘gene-low’ groups based on the mean value for the variable being analysed, which was provided in TCGA survival tool files. Kaplan–Meier curves were generated using R software, and between-group statistics were performed with a log-rank test. For the morphologic subclass analysis, M6 and M7 AML were excluded due to less than ten samples representing each group. Overall survival HRs were calculated for each cancer subset using Cox proportional hazard regression analysis.

### Beat AML data analysis

Inhibitor sensitivity (area under the curve, AUC) and *SDHA* or *SDHB* gene expression data were downloaded from vizome (https://www.vizome.org/aml/). Using R software, a for-Loop was used to perform regression analyses comparing gene expression level to inhibitor AUC. Data were expressed as a regression coefficient associated with inhibitor AUC and *SDHA* or *SDHB* expression levels, as well as an adjusted *P* value.

### Statistics and reproducibility

Experiments were designed to provide results that were easy to analyse and statistically robust. No statistical method was used to predetermine sample size, but a standard of at least *n* = 3–4 replicate experiments were used for in vitro studies, and *n* = 10 mice per group for animal studies. Data distribution was assumed to be normal, but this was not formally tested for all experiments. Data are presented as the mean ± s.e.m. for all experiments. No data were excluded from the analyses. Data collection and analysis were not performed blind to the conditions of the experiments. *P* values were calculated using a combination of methods including two-tailed *t*-tests, ANOVA and post hoc pairwise comparisons as indicated in figure legends.

### Reporting summary

Further information on research design is available in the Nature Portfolio Reporting Summary linked to this article.

## Supplementary information

Reporting Summary

Supplementary Tables 1–8

## Source data

Source Data Figs. 1–7 and Extended Data Figs. 1–7 Statistical source data.

## Data Availability

All data presented in this study are available in the main article and its Supplementary Information. Gene-effect data from Project Achilles were downloaded from the DepMap portal (https://depmap.org/). The Broad Institute has information on their website and the website dedicated to the Dependency Map project about how the raw data were generated and provides a list of references. Results from the pathway–pathway coessentiality are provided as a web application at https://www.datadrivenhypothesis.org/, which was developed by the Hirschey Lab. Source data are provided with this paper.

## Code availability

Code is available on GitHub (https://github.com/hirscheylab/ddh/) for a package we generated that contains functions to create plots and tables and https://github.com/hirscheylab/ddh_app/ for the web app.
