# Supplementary material for: Pathway coessentiality mapping reveals complex II is required for de novo purine biosynthesis in acute myeloid leukaemia
Source: Nat Metab. 2025 Dec 5;7(12):2474–88. doi: 10.1038/s42255-025-01410-x (PMC12727499; doi:10.1038/s42255-025-01410-x)
Supplement: Supplementary file 1 — Reporting Summary [file 42255_2025_1410_MOESM1_ESM.pdf]

nature portfolio

Corresponding author(s): Alexandre Puissant, Kris C. Wood and Matthew D. Hirschey

Last updated by author(s): Oct 15, 2025

Reporting Summary

Nature Portfolio wishes to improve the reproducibility of the work that we publish. This form provides structure for consistency and transparency in reporting. For further information on Nature Portfolio policies, see our Editorial Policies and the Editorial Policy Checklist.

Statistics

For all statistical analyses, confirm that the following items are present in the figure legend, table legend, main text, or Methods section.

n/a	Confirmed	
☐	☒	The exact sample size (*n*) for each experimental group/condition, given as a discrete number and unit of measurement
☐	☒	A statement on whether measurements were taken from distinct samples or whether the same sample was measured repeatedly
☐	☒	The statistical test(s) used AND whether they are one- or two-sided<br>*Only common tests should be described solely by name; describe more complex techniques in the Methods section.*
☐	☒	A description of all covariates tested
☐	☒	A description of any assumptions or corrections, such as tests of normality and adjustment for multiple comparisons
☐	☒	A full description of the statistical parameters including central tendency (e.g. means) or other basic estimates (e.g. regression coefficient) AND variation (e.g. standard deviation) or associated estimates of uncertainty (e.g. confidence intervals)
☐	☒	For null hypothesis testing, the test statistic (e.g. *F*, *t*, *r*) with confidence intervals, effect sizes, degrees of freedom and *P* value noted<br>*Give P values as exact values whenever suitable.*
☒	☐	For Bayesian analysis, information on the choice of priors and Markov chain Monte Carlo settings
☒	☐	For hierarchical and complex designs, identification of the appropriate level for tests and full reporting of outcomes
☒	☐	Estimates of effect sizes (e.g. Cohen's *d*, Pearson's *r*), indicating how they were calculated

Our web collection on statistics for biologists contains articles on many of the points above.

Software and code

Policy information about availability of computer code

Data collection	Cell viability and count data collected with DeNovix CellDrop FL, metabolomics data collected using Xcalibur 4.1 software and Tracefinder 4.1 software (ThermoFisher Scientific), proteomics collected with Proteome Discoverer 3.1 (PD3.1, ThermoFisher Scientific)
Data analysis	-Data analysis and Statistical tests were performed in GraphPad Prism 8-10, Microsoft Excel 365, MAGeCK, Proteome Discoverer, and in R.

For manuscripts utilizing custom algorithms or software that are central to the research but not yet described in published literature, software must be made available to editors and reviewers. We strongly encourage code deposition in a community repository (e.g. GitHub). See the Nature Portfolio guidelines for submitting code & software for further information.

Data

Policy information about availability of data

All manuscripts must include a data availability statement. This statement should provide the following information, where applicable:

- Accession codes, unique identifiers, or web links for publicly available datasets
- A description of any restrictions on data availability
- For clinical datasets or third party data, please ensure that the statement adheres to our policy

Data Availability
All data presented in this study are available in the main article, extended data, supplemental information or source files. Gene effect data from Project Achilles were downloaded from the DepMap portal at: depmap.org. The Broad Institute has information on their website and the website dedicated to the Dependency

Map project about how the raw data were generated and provides a list of references. Results from the pathway-pathway coessentiality are provided as a web application at www.datadrivenhypothesis.org, which was developed by the Hirschey Lab.

Research involving human participants, their data, or biological material

Policy information about studies with human participants or human data. See also policy information about sex, gender (identity/presentation), and sexual orientation and race, ethnicity and racism.

Reporting on sex and gender	N/A
Reporting on race, ethnicity, or other socially relevant groupings	N/A
Population characteristics	N/A
Recruitment	N/A
Ethics oversight	N/A

Note that full information on the approval of the study protocol must also be provided in the manuscript.

Field-specific reporting

Please select the one below that is the best fit for your research. If you are not sure, read the appropriate sections before making your selection.

☒ Life sciences ☐ Behavioural & social sciences ☐ Ecological, evolutionary & environmental sciences

For a reference copy of the document with all sections, see nature.com/documents/nr-reporting-summary-flat.pdf

Life sciences study design

All studies must disclose on these points even when the disclosure is negative.

Sample size	Samples sizes were at minimum n = 3, as indicated in figure legends. Sample sizes could sufficiently identify statistically significant changes.
Data exclusions	No data were excluded from analyses.
Replication	Experiments were reproduced in at least three independently conducted experiments (biologic replicates).
Randomization	Randomization was not required for any experiments.
Blinding	Blinding was not performed for in vitro experiments due to different culture conditions needed for treatment groups.

Reporting for specific materials, systems and methods

We require information from authors about some types of materials, experimental systems and methods used in many studies. Here, indicate whether each material, system or method listed is relevant to your study. If you are not sure if a list item applies to your research, read the appropriate section before selecting a response.

Materials & experimental systems

n/a	Involved in the study	
☒	☐	Antibodies
☐	☒	Eukaryotic cell lines
☒	☐	Palaeontology and archaeology
☐	☒	Animals and other organisms
☒	☐	Clinical data
☒	☐	Dual use research of concern
☒	☐	Plants

Methods

n/a	Involved in the study	
☒	☐	ChIP-seq
☒	☐	Flow cytometry
☒	☐	MRI-based neuroimaging

Eukaryotic cell lines

Policy information about cell lines and Sex and Gender in Research

Cell line source(s)	All cell lines were from American Type Culture Collection (ATCC) or Duke University Life Science Facility (LSF).
Authentication	All cell lines were authenticated prior to use using STR profiling.
Mycoplasma contamination	All cell lines were tested and confirmed negative for mycoplasma prior to their use.
Commonly misidentified lines (See ICLAC register)	The U118MG glioblastoma cell line is a commonly misidentified cell line by ICLAC, reportedly identical to the U138MG glioblastoma cell line. This cell line was included only in our complex II inhibitor viability screen as a representative glioblastoma

Animals and other research organisms

Policy information about studies involving animals; ARRIVE guidelines recommended for reporting animal research, and Sex and Gender in Research

Laboratory animals	MLL-AF9 syngeneic mice
Wild animals	none
Reporting on sex	both male and female mice were used
Field-collected samples	none
Ethics oversight	French National Ethics Committee on Animal Care (authorization number APAFIS 8909-2017021413452743 v1)

Note that full information on the approval of the study protocol must also be provided in the manuscript.

Plants

Seed stocks	N/A
Novel plant genotypes	N/A
Authentication	N/A
